# Tuberculosis care strategies and their economic consequences for patients: the missing link to end tuberculosis

**DOI:** 10.1186/s40249-016-0187-9

**Published:** 2016-11-01

**Authors:** Belete Getahun, Moges Wubie, Getiye Dejenu, Tsegahun Manyazewal

**Affiliations:** 1 Debre Markos University, P.O. Box 269, Debre Markos, Ethiopia; 2 Ethiopian Public Health Association, P.O. Box 7117, Addis Ababa, Ethiopia

**Keywords:** Tuberculosis, Directly observed therapy (DOT), Cost of TB, Out-of-pocket (OOP) payments, Loss income, Catastrophic cost, End TB, High-burden countries, Ethiopia

## Abstract

**Background:**

While investment in the development of Tuberculosis (TB) treatment strategies is essential, it cannot be assumed that the strategies are affordable for TB patients living in countries with high economic constraints. This study aimed to determine the economic consequences of directly observed therapy for TB patients.

**Methods:**

A cross-sectional cost-of-illness analysis was conducted between September to November 2015 among 576 randomly selected adult TB patients who were on directly observed treatment in 27 public health facilities in Addis Ababa, Ethiopia. Data were collected using interviewer-administered questionnaire adapted from the Tool to Estimate Patients’ Costs. Mean and median costs, reduction of productivity, and household expenditure of TB patients were calculated and ways of coping costs captured. Eta (η), Odds ratio and *p* values were used to measure association between variables.

**Results:**

Of the total 576 TB patients enrolled, 43 % were smear-positive pulmonary TB (PTB), 17 % smear-negative PTB, 37 % Extra-PTB and 3 % multi-drug resistant TB cases. Direct (Out-of-Pocket) mean and median costs of TB illness to patients were $123.0 (SD = 58.8) and $125.78 (*R* = 338.12), respectively, and indirect (loss income) mean and median costs were $54.26 (SD = 43.5) and $44.61 (*R* = 215.6), respectively. Mean and median total cost of TB illness to patient were $177.3 (SD = 78.7) and $177.1 (*R* = 461.8), respectively. The total cost had significant association with patient’s household income, residence, need for additional food, and primary income (*P* <0.05). Direct costs were catastrophic for 63 % of TB patients, regardless of significant difference between gender (*P* = 0.92) and type of TB cases (*P* = 0.37). TB patients mean productivity and income reduced by 37 and 10 %, respectively, compared with pre-treatment level, while mean household expenditure increased by 33 % and working hours reduced by 78 % due to TB illness. Income quartile categories were directly correlated with catastrophic costs (η = 0.684).

**Conclusion:**

Despite the availability of free-of-charge anti-TB drugs, TB patients were suffering from out-of-pocket payments with catastrophic consequences, which in turn were hampering the efforts to end TB. TB patients in resource-limited countries deserve integrated patient-centered care with comprehensive health insurance coverage, financial incentives, and nutrition support to reduce catastrophic costs and retain them in care. Such countries should induce home-based directly observed therapy programs to reduce costs due to attending health facilities, intensify home treatment of critically-ill patients with impaired mobility, and reduce the spread of TB due to patients traveling to seek care.

**Electronic supplementary material:**

The online version of this article (doi:10.1186/s40249-016-0187-9) contains supplementary material, which is available to authorized users.

## Multilingual abstract

Please see Additional file 1 for translations of the abstract into the six official working languages of the United Nations.

## Background

Integrated, patient-centered TB care and prevention is recognized as a major pillar in the post-2015 global TB strategy which envisions a world free of TB by 2035. The pillar’s core value is early detection, treatment and prevention of TB to ensure that all TB patients have equal, unhindered access to affordable services and are fully engaged in their care [1]. Although this pillar puts patients at the heart of service delivery, its implementation in High TB Burden Countries (HBC) is projected to demand complex resources and targeted efforts [2, 3]. TB care strategies in these settings have contended to deliver TB services free-of-charge, but economic challenges experienced by TB patients continued to hamper the system [4–6]. While implementation of the Directly Observed, Treatment Short course (DOTS) strategy (1994–2005) and the Stop TB Strategy (2006–2015) had significant contributions to the prevention and control of TB, they were also reported to pose a substantial economic burden on patients in terms of cost and affordability [7–10]. Out-of-Pocket (OOP) payments due to transportation, accommodation and food to get treatment at health facility aggravate economic crisis of TB patients: impacting their adherence to treatment [11–13] and forcing them to stop working, sell their properties, borrow money and reduce their overall income [14–17].

The initial milestone of the post-2015 global End TB strategy is to reduce TB deaths by 35 %, cut TB incidence rate by 20 %, and reduce TB-affected families facing catastrophic costs due to TB to 0 % between the years 2015 and 2020 [1]. Committed governments in HBCs, such as Ethiopia, which were able to achieve the Millennium Development Goal (MDG) 6 target of reducing the TB incidence rate, are also pledged to attain the post-2015 TB targets. In Ethiopia, the decline in incidence, prevalence and deaths related to TB [18] was an impressive gain in achieving the 2015 MDG 6 target. However, the country still ranks 3^rd^ in Africa and 10^th^ among the 22 HBCs in the world [19], and TB remains one of the leading causes of mortality [20]. Previous studies from different regions of Ethiopia reported that the cost of TB is a major barrier for patients to successfully complete care in Directly-Observed Therapy (DOT) [8, 21, 22].

Ensuring patient-centered TB care and prevention in HBCs ought to minimize financial burdens for TB patients and ratify that TB patients and affected families receive social protection interventions [6]. Understanding the direct and indirect costs of TB, their implications and costs of coping strategies for TB patients is essential to develop affordable TB care services in HBCs and to support poverty mitigation activities that could be grounded due to TB care strategies. This study was conducted in support of the integrated, patient-centered TB care and prevention pillar of the post-2015 TB strategy. It aimed to determine the economic consequences of the DOT strategy for TB patients and inform effective interventions required to successfully implement the end TB strategy in HBCs.

## Methods

A cross-sectional cost-of-illness analysis was conducted among adult TB patients who were attending TB care and treatment services.

### Study setting and participants

The study was conducted in public health facilities in Addis Ababa - the capital city administration of Ethiopia. The city administration had a total population of about 3.2 million [23] and a population density of 5 271 persons per Km^2^ [18]. The employment to population ratio was 45.86 while unemployment was 25 % [23]. Under the Addis Ababa city government, there were 96 public health facilities (90 health centers and six hospitals) of which 88 (92 %) were providing DOT services [24]. For this study, a list consisting of the 88 health facilities was received from the Addis Ababa City Government Health Bureau and 30 % (*n* = 27) of them were randomly selected using lottery method.

All TB patients who were detected as new cases in public and private health facilities in Addis Ababa (*n* = 7 298) were taken as the total population of the study. From this, all adult TB patients in the selected study facilities who were taking DOT services, at least for a month and not beyond the intensive phase of TB treatment (*n* = 1 673) were purposively selected as the study population to minimize recall bias. Sample size was calculated using single proportion formula [25], with 0.05 error allowance, 1.96 confidence level at 95 %, 0.05 level of catastrophic cost of OOP payments significance, 5 % contingency for non-response rate, and a design effect of 1.5. With this, the total sample size was calculated to be 604. Current TB registration books were reviewed at each study facility to determine the estimated TB caseloads and proportionally allocate the calculated sample site to each study facility.

### Data collection tool and techniques

Data were collected using interviewer-administered questionnaire comprising open- and close-ended questions in eight sections. The questionnaire was customized from a standardized tool to estimate TB patients’ costs developed by the World Health Organization (WHO), KNCV Tuberculosis Foundation, and the Japan Anti-Tuberculosis Association [26]. This tool was used to aid estimate the total costs of TB patients, guardian costs, reduction of productivity, and coping costs by adapting it to the local setting. The tool was translated to Amharic then backed to English.

The first section of the study questionnaire captured the “total costs” of TB illness to patient, in which direct and indirect medical (pre-diagnostic, diagnostic, and treatment) costs were included. In the first part of this section, items explaining the direct and indirect pre-diagnostic and diagnostic costs were captured to determine how much costs TB patients were incurred for each of their visits to health facility before they were identified as TB cases, including the visit when they actually received their diagnosis. The pre-diagnostic/diagnostic direct costs include costs for laboratory, X-ray, and drugs, while the indirect costs include costs for travels, food, accommodation, healthcare provider, administrative (consultative/registration), and for their time. This first part of this section also captured data to determine if insurance reimbursements whether applicable including reimbursed amount of the cost. In the second part of this section, items explaining the direct and indirect treatment costs were included. These costs had two components in the questionnaire: costs for ant-TB drug pick-up and costs for treatment follow-up. Costs for ant-TB drug pick-up was aimed to determine the frequency of travel, distance from facility, average length of stay, cost per round trip, cost of food per round trip, administrative costs, and accommodation costs of TB patients when taking anti-TB drugs. While costs for treatment follow-up was aimed to assess if TB patients were visiting health facility in addition to their regular visit schedules and determine associated costs. Costs for ant-TB medications were excluded from this category as this cost was fully covered by the Ethiopian government for all TB patients.

The second section of the questionnaire captured the total costs of guardians, which were for someone who accompanies the patient to the health facility or other visits because the TB patient cannot go by himself/herself. The questionnaire had captured data to determine if TB patients were going with guardians, and if applicable, the reason, how frequent, at which phase (pre-diagnostic/diagnostic/treatment), and how much a guardian was earnings per day.

The third section of the questionnaire was hospitalization cost which aimed to determine if TB patients had been hospitalized before or during their TB treatment and, if applicable, the length of stay and direct and indirect costs associated with it.

The fourth section was costs of food supplements for nutrition support to determine if TB patients were buying any supplements for their diet because of TB illnesses and, if applicable, what kind of items they have bought and who much money they spent.

The fifth section was health insurance. TB patients were asked if they have any kind of private or government health/medical insurance scheme and, if applicable, the type of insurance, and if reimbursements were made and the amount for any costs related to the TB illness which were to be cross-checked with the first section in the questionnaire.

The sixth section was coping costs, which were costs to meet daily requirements despite extra expenditures or loss of income, which include the sale of assets, taking up debt, saving on food or other items, taking a child out of school to care for the patient or taking up another job. TB patients were asked if they paid all the costs by themselves for TB illness, borrow any money to cover costs due to the illness and if yes how much and from whom and interest rate on the loan, ever leased anything, and sold any of their property to finance the cost of the TB illness and if yes the type of item with its market value and the actual earning from the sale of properties.

The seventh section was about income loss as well as income and affordability to TB treatment and healthcare. It had had multiple questions aimed at determining if TB patients stay away from work and loses income as a result of TB illness and estimate the total income loss due to TB illness and the impact costs had on patients.

The eighth section was for socioeconomic questions which aimed at capture TB patients’ data regarding the type of TB infection, dates TB treatment is started, total duration of planned treatment, treatment status, and socio-demographic status including average monthly income, occupation, household, and highest level of education. Household was referred to a family which consists of one or more people who live in the same dwelling and also share at meals or living accommodation.

The questionnaire was pre-tested with 27 TB patients who were representative of the study’s target group. After full informed consent obtained, TB patients were interviewed at study health facilities when they came to take their daily medications until the required number of participants were recruited at each facility. Ethical clearance to conduct this study was received from the institutional research ethics review committee of Debremarkos University and the Addis Ababa City Administration Health Bureau.

### Data analysis and definition of measurements

Data were entered into Statistical Packages for Social Science (SPSS), version 20, manufactured by IBM, Chicago, IL, USA to convert to electronic data. All the costs and income figures collected in Ethiopian currency (birr) were standardized to United State Dollar ($) with average exchange rate for the dates during which data were collected (birr 20.8979.8 = $1). Descriptive statistics such as mean, standard deviation (SD), median, range (R), and percentages were computed to explain the variables. *P* values, Eta (η), and Odds ratio were used as the measure of association between variables, where appropriate. One-way sensitivity analysis [27] was carried out on selected cost items to assess the variations in total costs of TB patients with their possible options. For regression analysis, the total median cost was used as a cut-off point to dichotomize the total cost into low and high payee. Inter-method regression model was used.

The “total cost” was calculated by addition of the entire “direct” and “indirect” costs of TB illness to patient. The “direct cost” was calculated by addition of any payment effected in relation to medical and non-medical costs. The “medical costs” were the sum of OOP payments for TB diagnosis and treatment made by TB patients in a given household (purchase of medicines, payments for diagnostic tests, net of reimbursements), while the “direct non-medical costs” were OOP payments related to the use of TB health services (payments for transport, lodging and food) before and during TB diagnosis and treatment. The “indirect costs” were TB patient or guardian lost income due to TB health-care seeking and hospitalization during the TB episode. It was calculated by multiplying the time that the patient did not work with the average individual take home earning before TB.

“Catastrophic costs” was calculated based on WHO’s explanation: whenever the out-of-pocket costs of TB illness are greater than or equal to 40 % of a TB patient’s household non-subsistence annual income [28] i.e. whenever the proportion of the ability to pay to out-of-pockets is greater or equal to 40 % with the consequence that the household suffer the burden of TB disease. Indirect costs of care and income loss are not included as WHO explains.

“Reduced productivity” was measured by calculating the difference in the actual performance of TB patients before and during TB illness. It was the number of hours a TB patient had been working per day when s/he was healthy minus the number of hours a TB patient was working per day when s/he falls to TB illness.

Regarding treatment phases, “pre-treatment” was the period of time from self-reported onset of TB-related symptoms until treatment initiation. “Intensive phase” was the first two consecutive months of TB treatment. “Continuation phase” was the four consecutive months immediately following the intensive treatment phase. “During treatment” was the period of time spanning from the beginning of the intensive treatment phase to the end of continuation treatment phases.

## Results

### Socio-demographic characteristics

Of the total 604 TB patients accessed, 576 (95 %) were responded to the questionnaire. Fifty-three percent were male, 90 % live in urban area, and 62.5 % employed, 18.1 % house wives and 11 % unemployed (Table 1). The minimum household monthly income was in the range $14.5–63.40 for 25 % of TB patients, while the maximum was in the range $148.83–335.00 for 25 % of TB patients.

**Table 1:** Socio-demographic characteristics of TB patients on DOT

Variable	Frequency	Percentage
Gender		
Male	304	52.8
Female	272	47.2
Age		
18–24	133	23.1
25–34	219	38.0
35–44	124	21.5
45–54	45	7.8
55–64	40	6.9
65 and above	15	2.6
Marital status		
Married	229	39.8
Single/never married	283	49.1
Widowed	45	7.8
Divorced	10	1.7
Separated	9	1.6
Residence		
Urban^a^	520	90.3
Rural^b^	54	9.4
Homeless^c^	2	0.3
No. of person in a household		
1–3	223	38.7
4–4	107	18.6
5–6	162	28.1
7–11	84	14.6
Highest educational level		
Further higher education	111	19.3
Secondary school, grade 9–12	243	42.2
Primary school, grade 5–8	147	25.5
Primary school, grade 1–4	22	3.8
No formal education	53	9.2
Ethnic group		
Oromo	136	23.6
Amhara	272	47.2
Tigre	64	11.1
SNNPR^d^	104	18.1
Household monthly income in $		
14.50–63.40	143	25.0
63.41–110.06	144	25.2
110.07–148.82	142	24.8
148.83–335.00	143	25.0
Occupation		
Permanent employee	97	16.8
Self-employee	149	25.9
Temporary employee	114	19.8
House wife	104	18.1
Unemployed	64	11.1
Pensioner	24	4.2
Student	24	4.2

*$* United States dollar; ^a^major town and cities; ^b^villages and hamlets; ^c^without formal house such as living on street; ^d^Gurage, Silte, Sidama

Based on their history of TB diagnosis, 248 (43 %) were smear-positive PTB cases, 96 (17 %) smear-negative PTB cases, 216 (37 %) E-PTB cases, and 16 (3 %) MDR-TB cases. Regarding their TB treatment outcome, 76 % were newly diagnosed, 17 % relapse, 3 % treatment failure, 3 % transfer in, and 1 % treatment default.

### Cost of TB

The direct (OOP) mean and median costs of TB illness to patients during DOT were $123.0 (SD = 58.8) and $125.78 (*R* = 338.12), respectively, and the indirect (loss income) mean and median costs were $54.26 (SD = 43.5) and $44.61 (*R* = 215.6), respectively. With these, the mean and median total costs of TB illness to patient during DOT were $177.3 (SD = 78.7) and $177.1 (*R* = 461.8), respectively (Table 2). Among the OOP, 37 % was for food supplements for nutrition support and 33.6 % was for hospital related direct costs.

**Table 2:** Cost of TB illness to patients under DOT

Costs of TB illness to patients	*N*	Mean (SD)	Median (R)
Direct costs (OOP)			
Pre diagnostic and diagnostic	576	24.71 (18.4)	20.10 (134.7)
Anti TB treatment follow up	576	23.42 (24.6)	22.97 (287.1)
Hospital admission related	96	66.04 (67.5)	44.26 (353.9)
Guardian/ accompany	388	1.49 (1.79)	0.96 (10.0)
Un scheduled additional follow up	104	8.36 (8.5)	5.70 (57.32)
Additional food	472	72.36 (46.7)	83.26 (201.0)
Direct cost total	**576**	**123.0 (58.8)**	**125.78 (338.12)**
Indirect costs (loss income)			
Pre diagnosis and diagnosis	360	14.31 (18.7)	11.32 (182.47)
Treatment follow up cost	360	41.04 (31.6)	30.51 (43.07)
Guardia/ accompany	388	5.19 (8.9)	0 (215.57)
Hospital stay	96	16.38 (15.8)	12.80(74.2)
Indirect costs total	**360**	**54.26 (43.5)**	**44.61 (215.6)**
Total cost (Direct + Indirect)	**576**	**177.30(78.7$)**	**177.14 (461.8)**

Figures are computed at Ethiopia average National Bank exchange rate of Birr 20.8979.8 to $1 in October 2015

*SD* Standard deviation, *R* Range

The total cost of TB illness to patients had significant association with patient’s household income, residence, need for additional food, and primary income (*P* <0.05) (Table 3).

**Table 3:** Factors associated with high cost of TB illness to patients under DOT

Variable	Total cost in USD		AOR	(95 % Confidence interval)		*P* value
	< 177.3 *n*	> 177.3 *n*		Lower boundary	Upper boundary	
Family income($)						0.033
< 117	162	139	1.847	1.051	3.248	
> 117	109	162	1.00			
Residence						<0.001
Urban	266	254	1.00	1.651	6.024	
Rural	18	36	3.154			
Additional food cost						<0.001
Yes	208	264	5.733	3.314	9.920	
No	78	26	1.00			
Primary income earner						0.010
Patient	157	167	3.876	1.382	10.872	
Wife/mom	8	12	6.884	1.732	27.357	
Husband/father	101	105	3.532	1.199	10.404	
Son/daughter	20	6	1.00			
Cost covered by						0.029
Patient and/or family	251	270	0.412	0.299	0.98	
Others (friends, neighbor)	35	20	1.00			

### Medical and non-medical cost

Mean medical and non-medical costs of TB illness to patients $18.9 (SD = 14) and $104 (SD = 55.5), respectively. The medical costs constituted only 15 % of the total direct cost, while the rest 85 % was for non-medical costs. The non-medical direct mean follow up costs for six month was $23 (SD = 24), out of which 73 % was disbursed for a daily follow up in the first two consecutive months from confirmation of TB (Table 4). After a patient is diagnosed as TB case, the direct mean treatment follow up costs was $23.5. Seventy-six percent of the direct mean direct treatment follow-up costs was for direct observations while a patient was taking TB medications at the health facility during an intensive phase; of which 68 % was incurred for food and water while a patient was travelling to the health facility and the rest 32 % was for transportation (Table 4).

**Table 4:** Direct treatment follow up costs of TB illness to patient under DOT

	Intensive phase			Continuation phase			Total phase (6 months)		
	Transport	Food & water	Total	Transport	Food & water	Total	Transport	Food & water	Total
Mean	5.38	11.79	17.18	1.96	4.29	6.25	7.34	9.67	23.42
SD	12.0	12.5	18.0	4.4	4.6	24.7	16.5	13.9	24.3
Median	4.21	10.53	16.84	1.53	3.83	6.13	5.74	7.65	22.97
Range	210.55	105.27	210.5	76.56	38.28	76.6	287.11	210.5	287.11

*SD* Standard deviation

### One-way sensitivity analysis

The total exclusion of additional food costs for nutrition support from the out-of-pocket cost could reduce the mean total costs of TB from $177.30 (SD = 78.7) to $104 (SD = 58.8). Reduction in frequency of treatment follow ups and avoidance of unscheduled follow ups also had substantial contributions in the reduction of OOP payments (Table 5).

**Table 5:** One-way sensitivity analysis of total costs of TB under DOT

	*n*	Mean (SD)	Median (R)
If additional food cost not considered, to be zero or food support practiced to TB patients			
Direct cost	576	50.60 (3.2)	45.85 (306.06)
Indirect cost	360	54.26 (43.5)	44.62 (215.6)
Total cost	576	104.95 (58.8)	92.70 (423.54)
If treatment follow-up phases are changed from every day to every week in intensive phase and from every week to every month for continuation phase			
Direct cost	576	104.26 (55.0)	107.40 (306.1)
Indirect cost	360	44.60 (6.0)	38.25 (215.9)
Total cost	576	148.82 (64.0)	145.65 (420.2)
No unscheduled follow-up considered, no additional follow-up cost			
Direct cost	576	115.70 (73.0)	120.78 (208.6)
Indirect cost	360	54.26 (42.3)	44.62 (215.6)
Total cost	576	169.96 (69.0)	165.50 (463.8)
If all TB patient diagnosis and treatment completed at out-patient department, without admission related cost			
Direct cost	576	110.00 (40.3)	112.60 (208.8)
Indirect cost	360	42.89 (8.2)	32.78 (203.3)
Total	576	136.00 (29.9)	145.18 (455.70)

### Catastrophic costs

Among the 576 TB patients in this study, OOP payments were catastrophic for 63 % of TB patients. The proportion incurring catastrophic costs did not have significance difference between gender (*P* = 0.92) and type of TB cases (*P* = 0.37). The proportion incurring catastrophic costs was significantly different across number of individuals in a household and across different household monthly income quartiles (*P* <0.001). The highest income quartile categories were directly correlated with reduction of catastrophic costs of OOP payments (η = 0.684). For TB patients with the highest household income quartile group, OOP payments was catastrophic only for 5.3 % of patients, whereas for those with the lowest household income quartile group it was catastrophic for 99.5 % of patients.

The study analyzed the association of different variables with catastrophic level of OOP payment and found no association between TB patients with different cases of TB, level of education, gender, went un-scheduled additional follow-up, presence of guardian, borrowed money, sold and leased assets (*P* >0.05). Whereas marginally significant associations of catastrophic costs were found between TB patients with different age groups (*P* = 0.056) and area of residence (*P* = 0.05) (Table 6).

**Table 6:** Factors associated with catastrophic costs of TB illness to patients

Variable	Catastrophic costs		AOR	(95 % Confidence interval)		*P* value
	Yes (n)	No (n)		Lower boundary	Upper boundary	
Additional food cost incurred						< 0.001
Yes	315	157	4.555	2.050	10.119	
No	46	58	1.00			
Borrowed						0.036
Yes	75	29	2.253	1.057	4.804	
No	286	186	1.00			
Monthly family income ($)						<0.001
< 117	275	26	48.038	22.581	74.190	
> 117	82	189	1.00			
Ever stopped working						< 0.001
Yes	231	107	1.918	1.351	2.721	
No	130	108	1.00			
Presence of care giver at home						< 0.001
Yes	45	50	0.430	0.274	0.677	
No	316	165	1.00			

### Coping costs

Only 56 (9.7 %) of TB patients had any kind of medical insurance scheme such as employee insurance for 24 (42.8 %) patients, community based health insurance for 8 (14.3 %) of patients, private health insurance for 16 (28.6 %) of patients and government-driven free treatment scheme for 8 (14.3 %) of patients. For majority (90 %) of TB patients, OOP payments were covered by their family members and for the rest 10 % by their neighbors and nearby friends. Among the total 576 TB patients, 18 % borrowed money to cope TB costs, of whom 34 % from neighbors, 26 % from friends, 23 % from relatives, and 17 % from organizations. None of them borrowed with interest. Eleven percent (11 %) of TB patients sold their properties to cope TB costs, and house utensils was the major properties TB patients were selling for coping costs.

### Implications

**Fig. 1:**
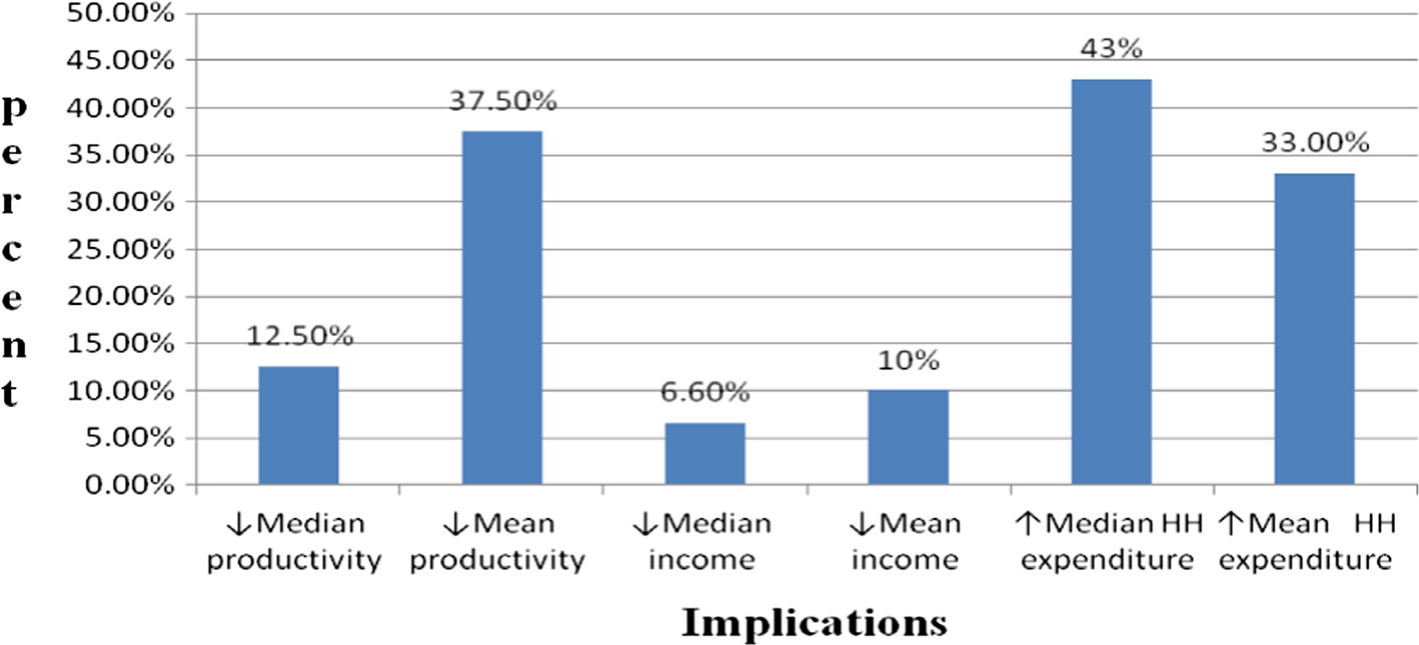
Implications of TB illness to patients

The mean and median drug collection time a TB patient spent per visit was 1.5 h (SD = 0.6 h) with a range of 3.5 h five times a week during intensive phase and once in a week for continuation phase of TB treatment. Among TB patients who were employees (*n* = 360), 78 % reduced working hours due to TB illness. Consequently, their mean productivity reduced by 37 % (SD = 22) and income by 10 % (SD = 30), and mean household expenditure increased by 33 % (SD = 25) (Fig. 1).

Among TB patients who were employees and/or students (*n* = 484), 240 (59.5 %) ever stopped working and/or going to school due to TB illness at least for a day, of whom 22 % gave up for more than 8–34 days and 8.5 % totally stopped working. One hundred twenty one (21.7 %) of guardians were accompanying TB patients to go to health facilities by quitting their income earning jobs, while 95 (16.5 %) of TB patients had care giver at home, of which 73 (77 %) were quitting their income earning jobs to provide care of the TB patient at home.

## Discussion

The Ethiopian FMoH has given due attention and priority to the treatment, prevention and control of TB with technical and financial support from partners in the field. Because of the development and implementation of countrywide strategies which fully aligned with the globally recommended Stop TB strategy, Ethiopia augmented ability to achieve all TB related millennium development goals; reduced the incidence by 44 %, prevalence by 50.5 % and deaths related to TB by 64 % from 1990 baseline [29]. However, the economic consequences of the various TB strategies for patients had been a debatable issue. The cost-of-illness analysis we conducted proves that TB patients living in central Ethiopia are facing multiple challenges due to the high cost of the DOT strategy, and the cost ($177.3) was even quite higher than the $25 estimated for PTB patients in Southern region [30] and the $53 estimated for PTB patients in 10 district areas of Tigray region [31] and in Ethiopia, though it was by far lower than the $847 estimated for low-income countries [6]. The differences might arise from different study settings, time of determination as cost is time-sensitive, and the type of TB cases included in the studies.

This study shows that the existing DOT strategy which requires frequent use of healthcare facilities for TB treatment has a significant impact on the total cost of illness to TB patients. The one-way sensitivity analysis reveals that there is a 15 % reduction of OOP payments if DOT schedule is rearranged from daily to weekly for intensive phase and from weekly to monthly for continuation phase. Studies from different countries, including Ethiopia, also revealed that DOT is one of the major factors that lead to high cost of TB illness to patients as long as travelling to a health facility is tied with transport and food costs while on the way [3, 17, 31–38]. The challenge could enforce TB patients to discontinue treatment and acquire MDR and XDR TB, even when they understand the consequences of this. This needs careful policy designs that TB patients deserve an integrated patient-centered care with financial incentives to retain them in care. Some studies conducted in Ethiopia also recommend community-based treatment of TB patients through health extension workers [30] and bringing services closer to patients [12] to reduce OOP payments and retain TB patients in care.

Similarly, the study illustrated that OOP payments were catastrophic to majority (63 %) of TB patients. The level of this catastrophic cost had a significant change across different income quartile groups (*P* <0.05), similar to another multi-center study conducted in Ghana, Viet Nam and the Dominican Republic [37]. The estimated costs for direct observation of patients at health facilities and for pre-diagnostic and diagnostic from the total direct cost of TB illness to patients was nearly similar to studies reported in Zambia [15] and Burkina Faso [33], but lower than the estimate in South Africa [34]. This study didn’t show association between hospitalization cost and total cost, while other studies held in different settings reported an association between the two as the major cause for the higher costs of TB treatment [30, 31].

This study shows that the economic consequence of TB illness to patients was beyond OOP payments. Income loss constituted 30 % of the total cost of TB illness to patients, which was quite lower than the 60 % reported for low and middle-income [6]. The difference could arise from the difference of study participants’ average monthly income between study participants of this study and middle and low income countries where the review assessed. Moreover, 38 % of this study participant was students and pensioners’ without income earning jobs. Furthermore, the implication was reflected to the guardian of TB patients, with a significant number of guardians quitted their income earning jobs to accompany TB patients and give care at home. There was also a subsequent reduction of productivity and increment of household expenditure due to TB illness to patients. These call for an urgent need of financial incentives for TB patients in Central Ethiopia to enforce adherence to treatment. Financial incentives proved to be effective in improving treatment completion and reducing default rates among TB patients [30, 36].

Despite other studies [3, 30], in this study, age, gender, and educational status were not associated with total and catastrophic costs of TB illness to patients. The factors associated with high total cost due to TB in were family income, place of residence, payer of the cost, primary income earner, cost payer and additional food cost. TB patients who had lower income were less likely to pay high cost for their TB illness when compared to TB patients with high income, reflecting that TB patients with low income required a more financial incentives and health insurance coverage to survive up with TB illness. Similar to a study reported from Nigeria [3], TB patients who came from rural were more likely to disburse high OOP payments compared to urban residents, and patients who covered OOP payments by themselves were less likely to pay high cost compared to those whose OOP payments were covered by friends or other relatives.

Among healthcare financing mechanism, the potential strategies in Ethiopia, which were in use to protect patients from catastrophic costs were community based and social health insurance schemes [39]. In this study, only 9.7 % of TB patients had health insurance scheme, and they were trying to cope TB costs through loan and selling and leasing household properties. This catastrophic cost plus the consistent rise in the cost of living in Ethiopia, urges for comprehensive health insurance coverage of TB patients, with optimal balance of resource allocation between prevention and treatment. The Essentials of implementing the end-TB strategy emphasizes that transitioning from “stopping TB” to “ending the TB epidemic” will call for major transformations in national TB control efforts [40]. To this end, TB patients in HBCs such as Ethiopia with lower income need financial protection which should go beyond cost coverage for ant-TB drugs for patients to withstand high costs of TB illness. Though locally effective solutions such as national stop TB partnership were found to be promising in HBCs such as India, global level policy advises on most of the important elements of the TB elimination efforts are required to meet the global target of Zero TB-affected families facing catastrophic costs due to TB and 95 % reduction of TB deaths by 2035.

The study had some limitations in that it did not analyze costs patients incurred after TB treatment completion time and did not analyze social cost that cannot be determined in monetary bases. The study didn’t make other measures of poverty, such as house material or assets which would provide additional information on the overall ability of TB patients to withstand catastrophic costs. Despite this, the study clearly shows the total cost of TB illness to patients, guardian costs, hospitalization costs, health insurance coverage, catastrophic costs, coping costs and implication of costs of TB illness to patients who were on TB treatment at health facilities under DOT strategy.

## Conclusion

From this study, TB patients were suffering from OOP payments with catastrophic consequences. Additional food cost and frequency of follow-up for directly-observed therapy constituted high proportion and exert strong drive to high cost. TB patients had limited financial protection which results in adverse consequences such as borrowing money, selling good, income loss, productivity drop and household expenditure escalation. The economic consequences were beyond TB patients and their family; it reached to neighbors, relatives, and the community as well. Thus, TB patients in resource-limited countries deserve integrated patient-centered care with comprehensive health insurance coverage, financial incentives and nutrition support to reduce catastrophic costs and retain them in care. Such countries should induce home-based TB care programs to reduce catastrophic costs due to attending health facility, intensify home treatment of critically-ill patients with impaired mobility, help patients engage in key aspects of their daily lives, and reduce the spread of TB due to patients traveling to seek TB care.

## Additional file

Additional file 1: Multilingual abstract in the six official working languages of the United Nations. (PDF 647 kb)

## Abbreviations

CSA: Central statistical agency
DOT: Directly observed therapy
DOTS: Directly observed treatment, short course
EFMoH: Ethiopian Federal Ministry of Health
EPHI: Ethiopian Public Health Institute
HBC: High-Burden Countries
MDG: Millennium development goals
MDR –TB: Multi drug resistant tuberculosis
OOP: Out-of-pocket
TB: Tuberculosis
WHO: World Health Organization
