# Supplementary material for: Tuberculosis care strategies and their economic consequences for patients: the missing link to end tuberculosis
Source: Infect Dis Poverty. 2016 Nov 1;5:93. doi: 10.1186/s40249-016-0187-9 (PMC5088676; doi:10.1186/s40249-016-0187-9)
Supplement: Additional file 1: — Multilingual abstract in the six official working languages of the United Nations. (PDF 647 kb) [file 40249_2016_187_MOESM1_ESM.pdf]

Translation of the abstract into the six official working languages of the United Nations

استراتيجيات رعاية مرضى السل وآثارها الاقتصادية على المرضى: الحلقة المفقودة لإنهاء السل

بيليت جيتاهون، موجيس ووبي، جيتي ديجينو ، زاجاهون مانيازيوال

الملخص

الخلفية: على الرغم من أن الاستثمار في تطوير استراتيجيات علاج السل (TB) أمر ضروري، فلا يمكن افتراض أن الاستراتيجيات في متناول مرضى السل الذين يعيشون في بلدان تعاني من قيود اقتصادية عالية. تهدف هذه الدراسة إلى تحديد الآثار الاقتصادية للعلاج الخاضع للمراقبة المباشرة لمرضى السل.

الطرق: أجري تحليل مستعرض لتكلفة المرض في الفترة ما بين سبتمبر إلى نوفمبر 2015 بين 576 تم اختيارهم عشوائيًا من مرضى السل البالغين والذين كانوا تحت المراقبة المباشرة للعلاج في 27 مرفق من مرافق الصحة العامة في أديس أبابا، إثيوبيا. وقد تم جمع البيانات باستخدام استبيان عن طريق المقابلة مقتبس من أداة لتقدير تكاليف المرضى. تم حساب التكاليف المتوسطة والوسطية، والحد من الإنتاجية، وإنفاق الأسرة لمرضى السل وتم التقاط سبل التغلب على التكاليف. استخدمت إيتا (η) ونسبة الصعاب وقيم P لقياس العلاقة بين المتغيرات.

النتائج: من إجمالي 576 مريضًا مسجلين بالسل، كان 43٪ سل رئوي مسحة إيجابية (PTB)، و 17% مسحة سلبية PTB، و 37٪ إضافي PTB ، و 3٪ حالات سل مقاومة للعقاقير المتعددة. كانت التكاليف المباشرة (من الجيب) الوسيطة والمتوسطة من مرض السل للمرضى 123 دولار أمريكي (SD = 58.8) و78،125 دولار أمريكي (R = 338.12)، على التوالي، وكانت التكاليف غير المباشرة (خسارة الدخل) الوسيطة والمتوسطة 54,26 دولار أمريكي (SD = 43.5) و 44,61 دولار أمريكي (R = 215.6)، على التوالي. وكانت التكلفة الإجمالية الوسيطة والمتوسطة لمرض السل على المريض 177,3 دولار أمريكي (SD = 78.7) و177,1 دولار أمريكي (R = 461.8)، على التوالي. كان للتكلفة الإجمالية ارتباط كبير مع دخل أسرة المريض، والسكن، والحاجة إلى المزيد من المواد الغذائية، والدخل الأساسي (P <0.05). كانت التكاليف المباشرة كارثية لـ 63% من مرضى السل، بغض النظر عن الاختلاف الكبير بين الجنسين (P = 0.92) ونوع حالات السل (P = 0.37). لقد انخفضت الإنتاجية والدخل المتوسط لمرضى السل بنسبة 37٪ و 10٪ على التوالي، مقارنة مع مستوى ما قبل العلاج، في حين ارتفع متوسط إنفاق الأسرة بنسبة 33٪ وانخفضت ساعات العمل بنسبة 78٪ بسبب مرض السل. وكانت فئات الدخل الربيعية مرتبطة مباشرة بالتكاليف الكارثية (η = 0.684).

الخلاصة: على الرغم من توافر أدوية مضادة للسل مجانية، فإن مرضى السل يعانون من مدفوعات من الجيب ذات عواقب كارثية، وهذا بدوره يعرقل الجهود المبذولة لإنهاء السل. يستحق مرضى السل في البلدان ذات الموارد المحدودة رعاية متكاملة تركز على المريض مع تغطية شاملة للتأمين الصحي، وحوافز مالية، ودعم غذائي لتقليل التكاليف الكارثية والاحتفاظ بهم في الرعاية. وينبغي لهذه الدول الحث على برامج العلاج الخاضعة للمراقبة المباشرة المنزلية للحد من التكاليف الناجمة عن الحضور للمرافق الصحية وتكثيف العلاج المنزلي للمرضى ذوي الحالات الخطيرة مع إعاقة حركية، والحد من انتشار مرض السل بسبب سفر المرضى للحصول على الرعاية.

Translated from English version into Arabic by Free bird, through

TRANSLATORS
WITHOUT BORDERS

肺结核治疗策略及其经济后果：消除肺结核的缺失环节

Belete Getahun, Moges Wubie, Getiye Dejenu, Tsegahun Manyazewal

摘要

引言：尽管针对肺结核治疗策略的研发投入是必要的，但不能认为这些肺结核治疗策略就一定适用于生活在经济条件有限国家的患者。本研究的目的是确定直接监督治疗结核病患者的经济后果。

方法：在 2015 年 9 月-11 月，在埃塞俄比亚首都亚的斯亚贝巴开展一项横断面疾病费用分析研究。在 27 个公共卫生机构随机选取的 576 名正在接受直接监督疗法的成年结核病患者。采用访谈式问卷调查收集数据。计算费用平均值和中位数、生产率降低、结核病患者家庭支出并获取应对费用的方法。采用 Eta (η)，优势比、*P* 值来衡量变量之间的联系。
结果：纳入研究的 576 名结核病患者中，43%的为痰检阳性，17%为痰检阴性，37%的为肺外结核病，3%的为多耐药结核病病例。结核病病例直接（自费）费用均值和中位数分别为$123.0 (SD=58.8) 和$125.78 (R=338.12)。间接（收入损失）费用均值和中位数分别为$54.26 (SD=43.5) 和$44.61 (R=215.6)。总的费用与患者家庭收入、居住地、额外食品的需求、主要收入显著相关（$P<0.05$）。直接费用对于 63%的结核病人来说是灾难性的，不同性别($P=0.92$)和结核病类型($P=0.37$)之间的费用支出差异无统计学意义。与治疗前相比，结核病患者平均生产率和收入分别降低了 37%和 10%，平均家庭支出增加了 33%，且工作时间因为疾病减少了 78%。四分位数收入类别与灾难性费用直接相关（η=0.684）。
结论：尽管抗结核药物是免费发放的，然而患者仍需承担灾难性自费项目的后果，这在一定程度上阻碍了消除结核病的努力。在资源有限的国家，结核病患者应接受在综合医疗保险覆盖下的以患者为中心的治疗，通过财政激励和营养支持降低灾难性的费用支出，促使患者坚持治疗。这类国家应引进以家庭为基础的直接监督治疗项目来降低患者到卫生机构治疗的费用，加强对行动不便的危重患者的家庭治疗，降低因求医问药导致的肺结核传播。

Translated from English version into Chinese by Xin-Yu Feng, edited by Pin Yang

CDC CHINESE CENTER FOR DISEASE CONTROL AND PREVENTION
NATIONAL INSTITUTE OF PARASITIC DISEASES

Stratégies de traitement de la tuberculose et leurs conséquences économiques pour les patients: le chaînon manquant pour éradiquer la tuberculose

Belete Getahun, Moges Wubie, Getiye Dejenu, Tsegahun Manyazewal

Résumé
Contexte: bien que les investissements dans le développement de stratégies de traitement de la tuberculose (TB) s'avèrent essentiels, on ne peut pas supposer que les stratégies soient abordables pour les patients atteints de TB et vivant dans des pays affrontant de graves contraintes économiques. La présente étude a pour objectif de déterminer les conséquences économiques du traitement à observation directe de patients atteints de TB.
Méthodes: une analyse transversale du coût de la maladie a été menée entre septembre et novembre 2015 parmi 576 patients adultes atteints de TB sélectionnés de manière aléatoire qui suivaient un traitement à observation directe auprès de l'un des 27 établissements de santé publique d'Addis Abeba, Éthiopie. Les données ont été collectées à l'aide d'un questionnaire administré par un interviewer adapté à partir de l'Outil d'estimation des coûts pour les patients. Les coûts moyens et médians, la réduction de la productivité et les dépenses du foyer de chaque patient atteint de TB ont été calculés et les manières de gérer les coûts ont été consignées. Les valeurs eta (η), et *P* ainsi que le rapport de cotes ont été utilisés pour mesurer l'association entre les variables.
Résultats: sur les 576 patients atteints de TB inclus à l'étude, 43 % étaient atteints d'une TB pulmonaire (TBP) à frottis positif, 17 % étaient atteints d'une TBP à frottis négatif, 37 % étaient atteints d'une TBP extra et la proportion de cas de TB multi-résistante représentait 3 %. Les coûts

directs (réglés par le patient) moyens et médians de la TB dont les patients devaient s’acquitter s’élevaient respectivement à 123 dollars (ET = 58,8) et 125,78 dollars (R = 338,12) et les coûts (perte de revenu) moyens et médians s’élevaient respectivement à 54,26 dollars (ET = 43,5) et 44,61 dollars (R = 215,6). Le coût total moyen et médian de la TB pour les patients s’élevaient respectivement à 177,30 dollars (ET = 78,7) et 177,10 dollars (R = 461,8). Le coût total était fortement associé au revenu du foyer du patient, à son lieu de résidence, à sa nécessité d’aliments supplémentaires et à ses revenus primaires (P<0,05). Les coûts directs étaient catastrophiques pour 63 % des patients atteints de TB, indépendamment de la différence significative entre le genre (P=0,92) et le type des cas de TB (P=0,37). La productivité et les revenus des patients atteints de TB ont été respectivement réduits de 37 % et 10 % par rapport au taux antérieur au traitement, tandis que les dépenses moyennes du foyer ont augmenté de 33 % et les heures de travail ont quant à elles diminué de 78 % en raison de la TB. Des catégories du quartile du revenu ont été directement corrélées avec les coûts catastrophiques (η=0,684).

Conclusion: malgré la disponibilité de médicaments anti-tuberculiques gratuits, les patients atteints de TB devaient régler des frais de leur propre poche, ce qui se traduisait par des conséquences catastrophiques et entravait les efforts d’éradication de la TB. Les patients atteints de la TB dans les pays aux ressources limitées méritent d’avoir accès à des soins intégrés et centrés sur le patient associés à une couverture santé exhaustive, des incitations financières et une assistance nutritionnelle pour réduire les coûts catastrophiques et leur permettre de ne pas quitter le parcours de soin. Ces pays doivent lancer des programmes thérapeutiques à observation directe à domicile afin de réduire les coûts générés par les consultations auprès d’établissements de soins de santé, intensifier le traitement à domicile de patients en état critique et à mobilité réduite et diminuer la propagation de la TB due aux déplacements des patients cherchant à bénéficier de soins.

Translated from English version into French by eric ragu, through

TRANSLATORS
WITHOUT BORDERS

Способы лечения туберкулеза и их экономические последствия для пациентов: недостающий элемент для устранения болезни

Белет Гетахун, Могес Вуби, Гетье Деджену, Цегахун Маньязевал

Краткое изложение

История вопроса: Хотя инвестиции в разработку способов лечения туберкулеза чрезвычайно важны, не следует предполагать, что эти способы будут финансово доступны больным туберкулезом, проживающим в странах с высокими экономическими ограничениями. Целью данного исследования является определение экономических последствий непосредственно наблюдаемого лечения для больных туберкулезом.

Методы: Был проведен перекрестный анализ стоимости болезни в период с сентября по ноябрь 2015 г. среди 576 произвольно выбранных взрослых пациентов с туберкулезом, лечение которых непосредственно наблюдалось в 27 общественных медицинских учреждениях г.

Аддис-Абеба (Эфиопия). Данные собирались с помощью раздаваемых опрашивающим вопросников, основанных на «Средстве оценки расходов пациента». Были рассчитаны среднее и срединное значения расходов, сокращения работоспособности и домашних расходов больных туберкулезом и зафиксированы способы адаптации. Для расчета соотношения переменных использовались значения эта (η), отношения шансов и P.

Результаты: Из 576 пациентов, 43% имели туберкулез легких с положительным мазком, 17% – туберкулез легких с отрицательным мазком, 37% – внелегочный туберкулез и 3% – случаи полирезистентного туберкулеза. Средние и срединные значения прямых (оплачиваемых из собственных средств) расходов, связанных с туберкулезом, были равны $123,0 (SD=58,8) и $125,78 (R=338,12) соответственно, а непрямых (потеря дохода) – $54,26 (SD=43,5) и $44,61 (R=215,6), соответственно. Среднее и срединное значение общей суммы расходов на пациента были равны $177,3 (SD=78,7) и $177,1 (R=461.8) соответственно. Общая сумма расходов была сильно связана с доходом семьи пациента, места проживания, потребности в дополнительном питании и основным доходом (P<0,05). Прямые расходы были катастрофическими для 63% больных туберкулезом вне зависимости от разницы пола (P=0,92) и видов туберкулеза (P=0,37). В результате заболевания туберкулезом средняя работоспособность и доход пациентов снизились на 37% и 10% соответственно по сравнению с уровнем до лечения, а средние семейные расходы повысились на 33%, а рабочие часы снизились на 78%. Квартильные категории дохода были напрямую связаны катастрофическими расходами (η=0,684).

Заключение: Несмотря на доступность бесплатных лекарств от туберкулеза, пациентам приходилось делать платежи из собственных средств с катастрофическими последствиями, что, в свою очередь, препятствовало излечению болезни. Больные туберкулезом в странах с ограниченными ресурсами заслуживают интегрированного, ориентированного на нужды пациента медицинского обслуживания с комплексным медицинским страхованием, финансовыми стимулами и поддержкой питания для снижения катастрофических расходов и прохождения полного курса лечения. В таких странах следует вводить программы лечения с непосредственным наблюдением на дому для сокращения расходов пациентов на посещение медицинских учреждений, усиления домашнего лечения критических больных с пониженной мобильностью и снижения передачи туберкулеза во время перемещения больных к месту медицинского обслуживания.

Translated from English version into Russian by Elena McDonnell, through

TRANSLATORS WITHOUT BORDERS

Estrategias de tratamiento para la tuberculosis y consecuencias económicas para los pacientes: el eslabón perdido para terminar con la tuberculosis

Belete Getahun, Moges Wubie, Getiye Dejenu, Tsegahun Manyazewal

Resumen

Antecedentes: Si bien es fundamental la inversión en el desarrollo de estrategias para el tratamiento de la tuberculosis (TB), no se puede asumir que las mismas están al alcance de los pacientes con TB que viven en países con limitaciones económicas. El objetivo del presente estudio fue determinar las consecuencias económicas de los tratamientos de observación directa para pacientes con TB.
Métodos: Entre septiembre y noviembre del año 2015 se llevó a cabo un estudio transversal de costo de la enfermedad en 576 pacientes adultos con TB seleccionados al azar, que recibían tratamiento de observación directa en 27 establecimientos asistenciales de salud pública en Addis Ababa, Etiopía. Se recolectó información mediante cuestionarios administrados por entrevistadores adaptados de la Herramienta para Estimar los Costos del Paciente. Se calcularon los costos mediano y promedio, la disminución en la productividad y el gasto por vivienda de los pacientes con TB y se capturaron los costos de los modos de afrontamiento. Para medir la relación entre las variables se utilizaron valores eta (η), razón de momios y valores *P*
Resultados: Del total de 576 pacientes con TB registrados, 43% fueron enfermos bacilíferos (PTB), 17% enfermos no bacilíferos, 37% Extra-PTB y 3% casos de TB resistente a múltiples drogas. Los costos mediano y promedio directos (de bolsillo) de la enfermedad de la TB para los pacientes fueron de \$123.0 (SD=58,8) y \$125.78 (R=338,12), respectivamente, y los costos mediano y promedio indirectos (pérdida de ingresos) fueron de \$54.26 (SD=43,5) y \$44.61 (R=215,6), respectivamente. El costo total mediano y promedio de la enfermedad de la TB para los pacientes fue de \$177.3 (SD=78,7) y \$177.1 (R=461,8), respectivamente. El costo total se vio significativamente asociado con el ingreso familiar del paciente, su residencia, la necesidad de alimentación adicional, y el ingreso primario ($P<0.05$). Los costos directos fueron catastróficos para 63% de los pacientes con TB, sin importar la diferencia significativa entre géneros ($P=0.92$) y tipos de casos de TB ($P=0.37$). La productividad y el ingreso promedio de los pacientes con TB se redujo en 37% y 10%, respectivamente, comparado con el nivel pre-tratamiento, mientras que el gasto familiar aumentó en un 33% y las horas trabajadas se redujeron en un 78% debido a la enfermedad de la TB. Las categorías de cuartiles de ingresos se vieron directamente asociadas con los costos catastróficos ($\eta=0.684$).
Conclusión: A pesar de la disponibilidad de medicamentos anti-TB gratuitos, los pacientes con TB tenían que realizar pagos de sus bolsillos con consecuencias catastróficas, lo que a su vez entorpecía los esfuerzos para terminar con la TB. Los pacientes con TB en países con recursos limitados merecen una atención integrada centrada en el paciente con cobertura médica integral, incentivos financieros, y apoyo nutricional para disminuir los costos catastróficos y hacer que los pacientes continúen con su atención. Dichos países deberían inducir programas de tratamiento de observación directa en el hogar para disminuir los costos asociados con el tener que acudir a establecimientos asistenciales, intensificar el tratamiento en el hogar para pacientes en estado crítico con trastornos de movilidad, y disminuir la propagación de la TB debido a que los pacientes tienen que trasladarse para recibir tratamiento.

Translated from English version into Spanish by Maria Alejandra Aguada, through

TRANSLATORS
WITHOUT BORDERS
